# EGNAS: an exhaustive DNA sequence design algorithm

**DOI:** 10.1186/1471-2105-13-138

**Published:** 2012-06-20

**Authors:** Alfred Kick, Martin Bönsch, Michael Mertig

**Affiliations:** 1 Professur für Physikalische Chemie, Mess- und Sensortechnik, Technische Universität Dresden, Dresden 01062, Germany; 2 Kurt-Schwabe-Institut für Mess- und Sensortechnik e.V. Meinsberg, Kurt-Schwabe-Straße 4, Ziegra-Knobelsdorf 04720, Germany

**Keywords:** DNA sequence design algorithm, Hairpin, stem-and-loop structure, Single nucleotide polymorphism (SNP), Single-base extension (SBE), Polymerase chain reaction (PCR), DNA computing, DNA origami

## Abstract

**Background:**

The molecular recognition based on the complementary base pairing of deoxyribonucleic acid (DNA) is the fundamental principle in the fields of genetics, DNA nanotechnology and DNA computing. We present an exhaustive DNA sequence design algorithm that allows to generate sets containing a maximum number of sequences with defined properties. EGNAS (Exhaustive Generation of Nucleic Acid Sequences) offers the possibility of controlling both interstrand and intrastrand properties. The guanine-cytosine content can be adjusted. Sequences can be forced to start and end with guanine or cytosine. This option reduces the risk of “fraying” of DNA strands. It is possible to limit cross hybridizations of a defined length, and to adjust the uniqueness of sequences. Self-complementarity and hairpin structures of certain length can be avoided. Sequences and subsequences can optionally be forbidden. Furthermore, sequences can be designed to have minimum interactions with predefined strands and neighboring sequences.

**Results:**

The algorithm is realized in a C++ program. TAG sequences can be generated and combined with primers for single-base extension reactions, which were described for multiplexed genotyping of single nucleotide polymorphisms. Thereby, possible foldback through intrastrand interaction of TAG-primer pairs can be limited. The design of sequences for specific attachment of molecular constructs to DNA origami is presented.

**Conclusions:**

We developed a new software tool called EGNAS for the design of unique nucleic acid sequences. The presented exhaustive algorithm allows to generate greater sets of sequences than with previous software and equal constraints. EGNAS is freely available for noncommercial use at http://www.chm.tu-dresden.de/pc6/EGNAS.

## Background

Deoxyribonucleic acid (DNA) has the remarkable ability of specific molecular recognition with regard to its sequence of the nucleic bases adenine (A), thymine (T), guanine (G) and cytosine (C). This sequence recognition is based on the Watson-Crick base pairing of complementary bases A-T and G-C [1].

However, in mixtures of many different DNA strands in solution or on surfaces a correct hybridization is crucial for most applications referring to genetics, DNA nanotechnology, DNA origami, and DNA computing [2-8]. Cross hybridizations have to be minimized by controlling the uniqueness of all possible subsequence motifs in the set of used sequences. Additionally, specific care has to be taken with regard to secondary structures that can occur by folding due to intrastrand interactions. Hairpin structures reduce the hybridization efficiency, the binding rates, and thus, the detection limits on DNA microarrays [9].

Here we describe the exhaustive DNA sequence design algorithm EGNAS (Exhaustive Generation of Nucleic Acid Sequences). This algorithm is realized in a C++ program and is used to generate sequences with controlled intra- and interstrand properties. EGNAS is compared with previous tools. Data from selected publications are reanalyzed by applying EGNAS to proof the viability of this new algorithm.

## Implementation

We realized the sequence design algorithm EGNAS in a program written in C++. It is currently a command line program that was compiled by GNU Compiler Collection for Linux, Mac OS X and Microsoft Windows operating systems. EGNAS is freely available for noncommercial use at http://www.chm.tu-dresden.de/pc6/EGNAS. A manual and the first version of EGNAS are attached as additional files [see Additional files 1, 2, 3 and 4].

For comparison of computing time, sequence generations were performed on one and the same computer system with Intel^*Ⓡ*^Core^TM^ i5 CPU 3.20 GHz and 4 GB RAM. The operating system was a 32-bit Ubuntu 10.04 (Linux). The intra- and interstrand properties were verified by the analysis option of the software Seed[10,11].

## Results and discussion

### Previous software and algorithms

Numerous strategies for the DNA sequence design are described in literature [2-4,10-25]. We do not intend to analyze all algorithms, but we evaluate our results directly by comparing with previously published data. Brenneman and Codon gave a brief overview to the topic of strand design [26]. To our best knowledge, so far, there is no software tool available offering the possibility to design oligonucleotide sequences with adequate consideration of intra- and interstrand interactions. Furthermore, a maximum set size of generated sequences is desirable for microarrays or DNA strands used as TAGs for addressing a high number of different targets.

Sets of sequences with defined properties can be achieved with the program Seed developed by Seiffert et al. [10,11], as well as with the software tools DNASequenceGenerator and CANADA by Feldkamp et al. [15,20,22,25]. These programs are freely available, work efficiently, provide satisfactory set sizes and meet the criteria of uniqueness among all sequences of a set concerning interstrand properties. However, it is difficult to achieve a big set size with these tools and concurrently to control intrastrand properties. It is hardly possible to avoid at the same time self-complementary subsequences, stem-loop structures and repetitions within one and the same strand, as these intrastrand properties can not be defined separately from the interstrand properties.

Both Seiffert and Feldkamp use the criton concept introduced by Seeman[2,3]. Critons are all *L*_*c *_bases long overlapping parts of a sequence strand. Their complements are termed anti-critons. Based on the criton rules, strands are generated consisting of unique basic sequences. There are $4^{L_{c}}$ critons and anti-critons. This fact limits the maximum possible number of valid sequences *N*_*s*_ with the length of *L*_*s*_ bases. Every sequence consists of (*L*_*s *_−*L*_*c*_ + 1) overlapping segments. To obey the criton rules, every basic sequence is used only once in the whole set and its complement is not allowed. If *L*_*c *_is even, one half of all possible sequences is complementary to the other half, and *N*_*s*_ can be estimated using

(1) $$N_{s}=\frac{4^{L_{c}}}{2(L_{s}-L_{c}+1)}.$$

If *L*_*c *_is odd, the maximum set size is estimated applying Equation (2), because there are $4^{\frac{L_{c}}{2}}$ self-complementary basic sequences that are not allowed.

(2) $$N_{s}=\frac{4^{L_{c}}-4^{\frac{L_{c}}{2}}}{2(L_{s}-L_{c}+1)}$$

Further limitations result from restrictions with respect to the guanine-cytosine content (GC content) and forbidden sequence motifs defined by the user.

**Figure 1:**
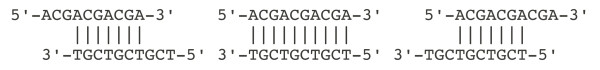
**Sliding.** Example for the sliding of a complementary sequence pair due to the repetition of 7 bases long subsequences.

### A novel sequence design algorithm

#### Sequence design criteria and options

The sequence design algorithm EGNAS offers the user different options. Consequently, the generated sequences meet certain criteria: 

1. Sequence length *L*_*s*_.

2. Length of basic sequences (criton length) *L*_*c*_.

3. Exact GC content or its range.

4. No terminal adenine or thymine in the strand./The demand on “GC ends”.

5. Forbidden sequences./Included sequences.

6. Length of forbidden self-complementary subsequences *L*_*sc*_.

7. Forbidden stem length of hairpin structures *L*_*hp*_.

8. Length of subsequences that are not allowed to be repeated within one and the same sequence (“sliding”) *L*_*sl*_.

9. Forbidden length of subsequences that could interact with complementary neighboring sequences *L*_*ni*_.

The EGNAS software provides the option to calculate the molar free enthalpy of DNA duplex formation. This calculation is based on the nearest-neighbor model [27] with parameters taken from SantaLucia et al. [28]. Marky et al. investigated the helix-to-coil transition and described the “fraying” of a DNA double strand at the terminal T-A base pairs [29]. In the nearest-neighbor model, SantaLucia et al. assigned a penalty of 0.4 kcal/mol for strands with a thymine base at the 5’ end [28]. Thus, with EGNAS it is possible to generate sequences that do not contain terminal adenine or thymine. The risk of fraying in the designed DNA strands can be reduced by only allowing guanine or cytosine at the terminal strand positions. We refer to this option as the demand on “GC ends”.

Guanine-rich motifs in DNA can form parallel four-stranded complexes [30]. Furthermore, it is known that telomeric ends of eukaryotic chromosomes contain guanine-rich overhangs and form intra- and intermolecular structures [31]. Therefore, the subsequences “GGG” or “CCC” are often forbidden in DNA strand design to circumvent the formation of guanine tetrads between hairpins.

**Figure 2:**
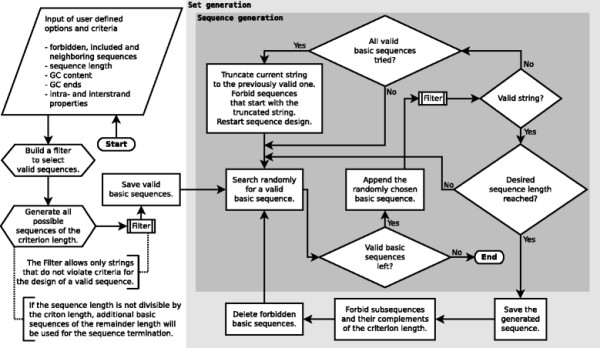
**Flowchart of the exhaustive design algorithm.** Simplified description of the exhaustive design algorithm of EGNAS. The input of the design criteria and options is used to generate basic sequences and the filter. The filter extracts valid basic sequences before the set generation. Additionally, strings are filtered within the sequence generation.

Homopolymeric runs of adenine or thymine are unwanted for certain applications requiring DNA polymerization. This process depends on the fidelity of DNA polymerases, which is influenced by the strand sequence that should be replicated. The repeat of one and the same base in the sequence can lead to error-prone replication through template-primer slippage [32]. Therefore, adenine or thymine runs, starting from four repeats, are preferentially forbidden for the sequence design. Forbidden sequences or subsequence motifs are specified by listing each of them in a set that is denoted further on in curly brackets. For instance, if both subsequences “GGG” and “CCC” are not allowed, the set containing the forbidden elements will be {*GGG*;*CCC*}.

#### Intrastrand properties

EGNAS offers novel options concerning the intrastrand properties. These are essential prerequisites to avoid secondary structures due to self-complementary sequences and hairpin formation. In contrast to the criton concept [2,3], as applied by Seiffert et al. [10,11] and Feldkamp et al. [15,20,22], the novel algorithm treats intrastrand properties separately. Below, we define the used terms and describe these properties.

##### Hairpins and self-complementarity

Hairpin structures are also called stem-and-loop structures. They consist of two complementary arm sequences and the loop sequence. The arm sequences are able to form the double-stranded stem while being connected by the single-stranded loop sequence. Self-complementarity is treated as a special case of a hairpin when the loop size is zero. Therefore, a self-complementary sequence has always an odd number of bases. If hairpin structures with a *L*_*hp *_bases long stem are forbidden, self-complementary subsequences that are equal to or longer than 2*L*_*hp*_ bases will consequentially be omitted.

##### Sliding

We use the term “sliding” for an intrastrand property of a sequence. Sliding denotes that a subsequence can be found several times at different positions of one and the same strand. If a complementary strand hybridizes with such a strand, different positions will be possible. Thus, sliding between the hybridizing strands would take place. For example, the sequence pair in Figure 1 allows sliding due to the repetition of 7 bases long subsequences (*L*_*sl *_= 8).

##### Interaction with the neighboring sequences

If primers are paired with TAGs, a special criterion for the TAG sequences will arise. In this case, primer foldback can become a problem. For example, the formation of hairpins will cause signals in single-base extension (SBE) reactions even lacking a template.

In the literature different approaches are given for the primer-TAG pairing. Those methods work with sets of previously found TAGs. Hirschhorn et al. suggested the calculation of an empirical foldback score [7]. Accordingly, if a foldback score is greater than a threshold value, the SBE primer will be paired with another TAG. Kaderali et al. used a free energy alignment algorithm for primer-TAG pairing [19].

We limit the interaction with neighboring strands already during the sequence generation. Therefore, all possible *L*_*ni*_ bases long complementary subsequences of a neighboring strand are forbidden for the generation of the corresponding TAG. This is especially intended for designing strands where a molecular spacer is located between the neighboring sequence and the TAG. For example, such a spacer could be a hexaethylene glycol moiety [9]. Actually, overlapping subsequences, which would evolve through directly attaching one neighboring sequence to either the 3’ or 5’ end of a TAG, are not considered. Nevertheless, in Section “Combination of TAGs with primers” we show that even if TAGs are attached to neighboring sequences without an intended spacer, hairpin formation will still be diminished significantly.

#### Description of the exhaustive design algorithm

In the following section we describe, how EGNAS provides a set of valid sequences. A simplified flowchart of the underlying algorithm is given in Figure 2. Initially, all *L*_*c*_ bases long subsequences in the included and neighboring sequences are read out and saved together with their complements as forbidden sequences. The included and neighboring sequences are predefined by the user, meaning that *L*_*c *_bases long cross-hybridizations are not allowed to occur between one of them and any of the generated sequences. Subsequently, all possible basic sequences with the length *L*_*c*_ are generated in such a way that they obey the criteria specified by the user. Thereafter, a string is formed stepwise from left to right by randomly combining allowed basic sequences. If the sequence length is not divisible by the criton length, additional basic sequences of the remainder length will be generated and used for the termination of the sequence design. After each step, the growing sequence is checked against the criteria chosen by the user.

**Figure 3:**
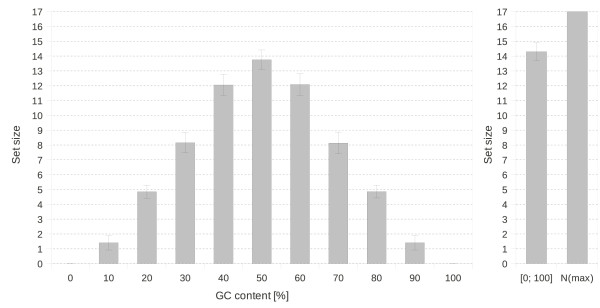
**Influence of the GC content and GC ends on the set size.** Dependence of the set sizes on the GC content. Sets of 10 bases long sequences with *L*_*c *_= 4 for global criton rules were generated. Averages ± standard deviations were calculated from 1,000 sets for restricted and from 10,000 sets for unrestricted conditions [0; 100]. The maximum set size *N*(*max*) = 17 is shown.

After trying all of the valid basic sequences as suffixes, there can still be combinations of basic sequences that do not allow appending of any remaining basic sequence, because the forming strings do not meet at least one of the necessary criteria. In such a case, this combination is forbidden to occur at the beginning of a string. Thus, a new trail will skip the basic sequences leading to the forbidden ones.

A sequence will be saved, if it has the defined length and meets all criteria. Every *L*_*c*_ bases long subsequence and its complement within this generated strand are forbidden and removed from the list of basic sequences. Then, the next sequence generation starts by the analog combination of the remaining basic sequences.

If all basic sequences are either forbidden or used, the sequence generation of the current set will be finished. Furthermore, the user can force every set to be complete. Namely, no additional sequences exist with the same user-defined restrictions of the set. The generation of a complete set is at the expense of computing time. Hence, limiting the maximum number of generated sequences can reduce the computing time significantly.

### Software comparison and performance tests

#### Global criton rules and intrastrand properties

In order to compare the novel algorithm with other available software tools, it was necessary to distinguish between two different settings of options for the sequence generation. On the one hand, the preassigned criton rules in Section “Intrastrand properties” are valid for the whole set of generated sequences. This holds for Seed[10,11], CANADA[25] and DNASequenceGenerator[15,20]. In this cases, we refer to *“global criton rules”*. It means for the intrastrand properties (*L*_*sc*_, *L*_*sl *_and *L*_*hp*_that they are treated equally to interstrand criteria. In detail, if the criton length is *L*_*c*_, *L*_*sc *_bases long self-complementary subsequences will be forbidden, as well as sliding of *L*_*sl *_bases long subsequences. Hairpin structures with a stem lengths of *L*_*hp *_bases and longer will not be allowed. Thereby, the following relations hold: 

1. *L*_*c *_=* L*_*sl *_=* L*_*hp*_

2. If *L*_*c *_is odd: *L*_*sc *_=* L*_*c*_

3. If *L*_*c *_is even: *L*_*sc *_=* L*_*c*_ + 1

On the other hand, EGNAS offers a control of intrastrand properties independent of the criton rules. In this case, the criton rules only control the cross hybridization among different sequences not taking into account self-complementarity, hairpins or sliding.

#### Influence of the GC content and GC ends on the set size

The influence of the GC content and GC ends were investigated by generating sets of sequences with global criton rules, *L*_*c *_= 4. Those sets were generated for every possible GC content of 10 bases long sequences.

The restriction of the GC content can lead to a reduced set size as shown in Figure 3. While 50% GC content is not necessarily a restriction to the set size, extreme values of the GC content limit the set size significantly. At GC contents of 30% and 70%, the set size is almost half compared with the set sizes achieved without restrictions or with 50% GC content.

The demand on GC ends in combination with the restriction of the GC content to exactly 50% lowers the set size significantly. Otherwise, no evident reduction of the set size is observed. With Equation (2), the maximum number of sequences is 17. With 16 sequences a yield of 94% could be achieved where no restrictions were set or only the GC content was forced to be 50%. These are high set sizes in relation to other calculations performed by Feldkamp[22] and those that are presented in Table 1.

**Table 1:** Influence of the criton and sequence length on the set size

**Sequence**	**Criton length**							
**length**	**4**		**5**		**6**		**7**	
10	14.2 ± 0.6	(17)	73.8 ± 1.2	(85)	348.9 ± 2.4	(403)	1,802.6 ± 7.6	(2,048)
11	12.7 ± 0.7	(15)	63.8 ± 0.9	(73)	289.8 ± 2.8	(336)	1,426.1 ± 8.7	(1,638)
12	11.4 ± 0.5	(13)	54.4 ± 0.7	(64)	246.2 ± 3.2	(288)	1,183.2 ± 4.0	(1,365)
13	9.7 ± 0.5	(12)	48.8 ± 1.0	(56)	214.4 ± 3.0	(252)	1,008.4 ± 4.9	(1,170)
14	8.9 ± 0.6	(10)	43.9 ± 0.6	(51)	190.9 ± 1.9	(224)	876.8 ± 2.9	(1,024)
15	8.1 ± 0.3	(10)	40.0 ± 0.5	(46)	172.1 ± 1.7	(201)	784.0 ± 1.9	(910)
16	7.7 ± 0.5	(9)	36.4 ± 1.0	(42)	155.6 ± 1.3	(183)	704.2 ± 3.7	(819)
17	7.0 ± 0.0	(8)	34.3 ± 0.8	(39)	142.5 ± 1.3	(168)	639.8 ± 3.3	(744)
18	6.4 ± 0.5	(8)	31.2 ± 0.4	(36)	131.2 ± 1.8	(155)	585.9 ± 3.6	(682)
19	5.9 ± 0.3	(7)	28.9 ± 0.6	(34)	122.5 ± 0.8	(144)	542.6 ± 4.2	(630)
20	5.9 ± 0.3	(7)	27.6 ± 0.8	(32)	113.8 ± 1.6	(134)	502.8 ± 2.3	(585)
21	5.6 ± 0.5	(6)	25.8 ± 0.4	(30)	106.5 ± 1.3	(126)	469.0 ± 2.9	(546)
22	5.0 ± 0.0	(6)	24.8 ± 0.6	(28)	100.1 ± 1.0	(118)	442.7 ± 2.9	(512)
23	5.0 ± 0.0	(6)	23.3 ± 0.5	(26)	94.9 ± 1.2	(112)	416.0 ± 1.9	(481)
24	4.6 ± 0.5	(5)	21.9 ± 0.6	(25)	89.6 ± 0.7	(106)	392.9 ± 2.1	(455)
25	4.1 ± 0.3	(5)	20.8 ± 0.4	(24)	84.8 ± 1.3	(100)	372.3 ± 1.3	(431)
26	4.0 ± 0.0	(5)	19.9 ± 0.3	(23)	80.8 ± 1.2	(96)	353.8 ± 1.5	(409)
27	4.0 ± 0.0	(5)	19.3 ± 0.5	(22)	77.2 ± 1.1	(91)	338.3 ± 1.6	(390)
28	4.0 ± 0.0	(4)	18.3 ± 0.5	(21)	73.8 ± 1.2	(87)	320.9 ± 1.5	(372)
29	4.0 ± 0.0	(4)	17.7 ± 0.5	(20)	70.7 ± 1.2	(84)	308.1 ± 1.7	(356)
30	3.9 ± 0.3	(4)	16.9 ± 0.6	(19)	68.4 ± 1.6	(80)	296.4 ± 1.8	(341)
31	3.1 ± 0.3	(4)	16.4 ± 0.5	(18)	65.3 ± 0.7	(77)	285.2 ± 2.2	(327)
32	3.0 ± 0.0	(4)	15.8 ± 0.4	(18)	63.5 ± 0.5	(74)	272.8 ± 2.0	(315)
33	3.0 ± 0.0	(4)	15.2 ± 0.4	(17)	60.6 ± 0.8	(72)	263.7 ± 1.6	(303)
34	3.0 ± 0.0	(3)	14.7 ± 0.5	(17)	58.7 ± 0.5	(69)	255.3 ± 1.6	(292)
35	3.0 ± 0.0	(3)	14.3 ± 0.5	(16)	56.6 ± 0.5	(67)	246.0 ± 1.5	(282)
36	3.0 ± 0.0	(3)	13.9 ± 0.3	(16)	54.7 ± 0.5	(65)	238.8 ± 1.5	(273)
37	3.0 ± 0.0	(3)	13.6 ± 0.5	(15)	53.3 ± 0.8	(63)	230.9 ± 1.2	(264)
38	3.0 ± 0.0	(3)	13.0 ± 0.0	(15)	51.7 ± 0.5	(61)	223.7 ± 0.9	(256)
39	3.0 ± 0.0	(3)	12.8 ± 0.4	(14)	50.1 ± 0.7	(59)	217.1 ± 2.0	(248)
40	3.0 ± 0.0	(3)	12.3 ± 0.5	(14)	48.8 ± 0.6	(57)	210.6 ± 1.7	(240)

Set sizes dependent on sequence and criton length for global criton rules. Averages ± standard deviations from 10 sets. The maximum possible set size in parentheses.

#### Influence of the criton and sequence length on the set size

Table 1 shows the dependence of the set size from the combination of sequence and criton length. For each of the 124 combinations, 10 sets were calculated with *L*_*c *_for global criton rules. In accordance with Equations (1) and (2), the set sizes grow with higher criton length and lower sequence length. Of course, the criton length has the highest influence, as it is in the exponent of those equations. Feldkamp presented an analog table [22].

In average, Feldkamp generated 84.4 ± 4.3% of the maximum possible numbers of sequences per set. Our new approach has a slightly higher average yield of 87.0 ± 4.6%. In 120 of the 124 different combinations of criton and sequence lengths, examined in Table 1, set sizes equal to or higher than those of Feldkamp’s tool were calculated with EGNAS.

#### Variation of the set size

**Figure 4:**
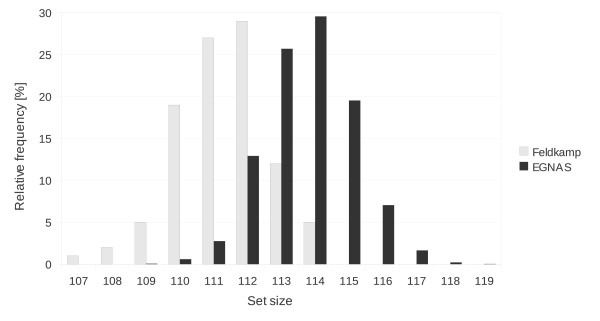
**Variation of the set size.** Histogram of the relative frequency of the set sizes. The results of EGNAS and Feldkamp[22] are compared with each other 10,000 sets of 20 bases long sequences with *L*_*c *_= 6 for a global criton rule were generated.

Another interesting issue of sequence sets is, how their sizes will be distributed, if the same options are chosen for multiple sequence generations. Figure 4 compares the results of Feldkamp’s [22] and of our software. The combination of *L*_*c *_= 6 and 20 bases long sequences was chosen for global criton rules. Feldkamp generated 100 sets with these settings. However, we evaluated 10,000 sets to gain a higher confidence level. The most of Feldkamp’s sets have 112 sequences, whereas the most of our completed sets contain 114 sequences. In both cases, these set sizes appear with a relative frequency of about 30%.

#### Sliding and its influence on the set size

**Figure 5:**
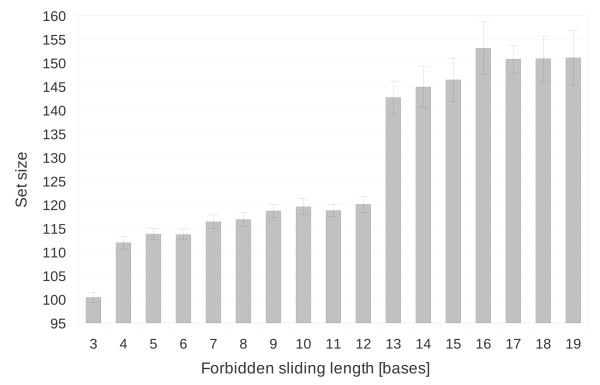
**Sliding and its influence on the set size.** Dependence of the set size on the sliding length (*L*_*sl*_). The sequence length is 20 and *L*_*c *_= 6 was used for global criton rules but with varying *L*_*sl*_. Averages ± standard deviation of 10 sets were calculated for each *L*_*sl*_.

Within one and same strand, EGNAS can control the repetition of subsequences of a certain length. As an example, we generated sets of sequences with *L*_*c *_= 6 for global criton rules but with varying *L*_*sl*_. The results of this calculations are shown in Figure 5. For *L*_*sl *_= 6, the global criton rules are valid, and in average, 113.8 ± 1.1 sequences are generated. Significant reduction of the set size to 100.4 ± 1.1 sequences is observable for *L*_*sl *_= 3. This is because the decrease of *L*_*sl *_reduces the number of usable basic sequences. A considerable increase of the set size to 142.7 ± 3.5 sequences is obtained by setting *L*_*sl *_= 13. In this case, *L*_*sl *_is large enough to allow the repetition of one basic sequence. Thus, for a complete strand, less basic sequences are used, and consequently, more usable basic sequences are left for the generation of further strands.

#### Controlling intrastrand properties

Self-complementarity and hairpins are the major reasons for the formation of secondary structures. Such structure formation competes against the hybridization with target DNA strands. Hence, a fast and sensitive detection on microarrays is hindered by secondary structures [9]. Accordingly, we generated sequences not only with minimum criton length *L*_*c *_for a global criton rule but we also prevented self-complementarity and the risk of hairpin formation. The DINAMelt Web Server was used to estimate the stability of secondary structures [33] ( http://mfold.rna.albany.edu/?q=DINAMelt/Two-state-folding (February 1, 2012)). The stabilities were calculated as the molar free enthalpies (Δ*G*) of the most stable hairpins at 37°C and 1 mol/l sodium ions.

Feldkamp et al. had generated 22 bases long sequences with the uniqueness *L*_*c *_= 5 and chose sequences with the most unstable secondary structures afterwards [34]. In Table 2 the generated sequences of Feldkamp et al. and our program are shown to compare the stabilities of possible secondary structures. Hairpins could be avoided by choosing *L*_*hp *_= 2. Even so, enough sequences could be generated to compete with Feldkamp’s sequence set. Feldkamp’s software tool is not able to treat intrastrand properties separately. For instance, a stem length of 4 bases can be found in sequence No. 11 (5'‐CAAGgtctgCTTG.....‐3').

**Table 2:** Avoiding hairpin formation in 22 bases long sequences

**No.**	**Δ*G***	**Feldkamp**	**Δ*G***	**EGNAS**
	$\left[\frac{\mathrm{kcal}}{\mathrm{mol}}\right]$	**5’ → 3’**	$\left[\frac{\mathrm{kcal}}{\mathrm{mol}}\right]$	**5’ → 3’**
1	-0.3	CCTGCGTCGTTTAAGGAAGTAC	*	GCTCATTTTACACTCTCCACCG
2	0.6	CAGCCAAGATTCTTTTACCGCC	*	CACACGGAGGCACAGAATAAAC
3	0.4	CCATCATGTGTGCCGAGATATG	*	GAACAGCGAAGAGATAGGAAGG
4	0.3	CTTCTCCTAACTGCACGGAATG	*	CCTTACTCGCCTTTCACATTCC
5	-0.3	GGTCCGGTCATAAAGCGATAAG	*	CAACTCACGCCACTACATCAAC
6	0.7	GTCCTCGCCTAGTGTTTCATTG	2.4	CAAGCCGTCAATAGTCCAAGTC
7	0.2	GGATCTGGCGCATAGACAATTC	2.1	CTGCTGAACCTGATACCGAAAC
8	-0.1	CACGTCACTGTTAATCCGAAGC	1.8	CAGTATTTCCAGTCAGTTCCGC
9	-0.1	GTGGAAAGTGGCAATCGTGAAG	1.8	CCTGTCGTTTTCTATGCTCCTG
10	0.6	GGACGAATACAAAGGCTACACG	1.8	CCTGCCGATGACCTACTTTTTG
11	-2.4	CAAGGTCTGCTTGATTTGGAGG	1.6	CCGTTCTTTGTCCTTGCTTCTC
12	-0.9	GTTTTGAACGTAGTAGAGCCGG	1.5	GTGATTGGCTGGTGTTGGTTTG
13	-0.2	GTAGGTGTCGGTGCGAAATTAG	1.3	GCTCGTGGTCTTGTTATGTCTG
14	-0.1	CTAGAACCGTTACGAGTTTGCG	1.1	GTAGATTTGAGGTGCGTTGTGG
*L*_*c*_		5		5
*L*_*hp*_		5		2
*L*_*sc*_		6		4
*L*_*sl*_		5		5

Comparison with Feldkamp et al. [34]. Comparison of 22 bases long sequences generated by Feldkamp et al. [34] and with EGNAS. The subsequences {*GGG;CCC*} were forbidden. GC ends were demanded. Molar free enthalpies of the most stable hairpins.

^*^No hairpin structure possible.

The restriction of *L*_*hp *_will improve sequence quality significantly, if no strand folding is desired. However, smaller values for *L*_*hp *_result in smaller set sizes. Calculations of 10 sets were performed with varying *L*_*hp*_ and *L*_*c *_=* L*_*sc *_=* L*_*sl *_= 6 for 20 bases long sequences. A clear reduction of the set size can be found from 111.6 ± 1.3 (*L*_*hp *_= 3) to 61.8 ± 1.2 (*L*_*hp *_= 2). For *L*_*hp *_= 6, the set size is 113.8 ± 1.5 and not greater than the set sizes with *L*_*hp *_= 4 or 5.

Further examples for preventing hairpins are given in Tables 3, 4 and 5. Tanaka et al. [17] and Feldkamp et al. [20] published sets of 20 bases long sequences that we compare with our results with respect to the stabilities of hairpin structures (Table 3). Sequences with lower stabilities of hairpins were designed by applying EGNAS. Additionally, the set size could be increased from 14 to 16 sequences.

**Table 3:** Avoiding hairpin formation in 20 bases long sequences

**No.**	**Δ*G***	**Tanaka et al.**	**Δ*G***	**Feldkamp**	**Δ*G***	**EGNAS**
	$\left[\frac{\mathrm{kcal}}{\mathrm{mol}}\right]$	**5’ → 3’**	$\left[\frac{\mathrm{kcal}}{\mathrm{mol}}\right]$	**5’ → 3’**	$\left[\frac{\mathrm{kcal}}{\mathrm{mol}}\right]$	**5’ → 3’**
1	1.1	ATGTACGTGAGATGCAGCAG	1.0	TAGTCGCGTGATTTGGAAGG	*	GAGAAGGAACACGATACAGC
2	1.1	ATCACTACTCGCTCGTCACT	0.9	GCTGTCTTTCGTCAATACCG	*	CCTTACACATTTCTTCCGCC
3	0.6	AGATGATCAGCAGCGACACT	0.3	CTGAACGGAATCTAGTAGCG	*	CACAATCAACTCTACCGCTC
4	0.5	TCTGTACTGCTGACTCGAGT	-0.1	GTCTACGGTTCTCTTACGCT	2.4	CCTGTCCTATCTTTCGCTTC
5	0.3	ACATCGACACTACTACGCAC	-0.2	AAAGCCGTCGTTTAAGGAGC	2.1	CTGGCTATGGAAACTGAACG
6	-0.2	GCTGACATAGAGTGCGATAC	-0.2	AATCGCAGTACAGATGGTGG	1.8	CTCGGTCTAAATCTGCTCTC
7	-0.5	TGTGCTCGTCTCTGCATACT	-0.2	GGATGACCAGAGCACTTCAA	1.8	GCCGTTATCCTCTGTTTGTC
8	-0.5	TCAGAGATACTCACGTCACG	-0.6	TACGTCTCGAACTGATAGCC	1.8	GGTTTATTGAGGTTGCGAGG
9	-0.8	CGAGTAGTCACACGATGAGA	-0.8	TGATCTTGTAAAGGCCAGGC	1.8	CCTCCGTATTTGCCTTGTTG
10	-0.9	CGAGACATCGTGCATATCGT	-0.8	TACGATACTTGGCGAGCCAT	1.5	GTTGTAGTTCGTTGGTGGTC
11	-1.2	AGACGAGTCGTACAGTACAG	-0.9	TGCAGAAAAACTATGCCGCC	1.5	CTTCGGCTGGTTCTATTCTG
12	-1.8	TATAGCACGAGTGCGCGTAT	-1.1	GCGCGGACAATTCATTGGTT	1.4	GGCTCACTCATCACACTAAC
13	-2.2	GATCTACGATCATGAGAGCG	-1.7	CCGCAATCCGGTGAAATTAG	1.4	CCTTTACGCCTGACTTTGAC
14	-2.3	GACAGAGCTATCAGCTACTG	-3.0	CTTAGGCAGGTGCCACATAT	1.3	CCTGCGTCTTATGTCTCTTG
15					0.9	GCGTGAATGAAGTGGAGTAG
16					0.9	GGATTACTTGCTTGGACTGG
						**GC ends**
				**#**		**§**
*L*_*c*_		9		5		5
*L*_*hp*_		5		5		2
*L*_*sc*_		10		6		4
*L*_*sl*_		7		5		5

Comparison with Tanaka et al. [17]Feldkamp et al. [20]. Comparison of 20 bases long sequences generated by Tanaka et al. [17], Feldkamp et al. [20] and EGNAS. Molar free enthalpies of the most stable hairpins.

^*^No hairpin structure possible.

^#^Without {*GGG;CCC*}.

^§^Without {*GGG;CCC;AAAA;TTTT*}.

**Table 4:** Calculations with included sequences to design additional 20 bases long sequences

**No.**	**Δ*G***	**Arita et al.**	**Δ*G***	**Feldkamp**	**EGNAS**
	$\left[\frac{\mathrm{kcal}}{\mathrm{mol}}\right]$	**5’ → 3’**	$\left[\frac{\mathrm{kcal}}{\mathrm{mol}}\right]$		**5’ → 3’**
1	2.5	CCGTCTTCTTCTGCT	1.5	AAAGCCGTCGTTTCC	GAGAGAAACGGCAAC
2	*	TTCCCTCCCTCTCTT	1.0	TTGTGGTACTCTGCG	CGCAAACTCACCTAC
3	3.0	CGTCCTCCTCTTGTT	1.1	TATTAGATGGCCGCC	GCCTTTACATCTCCG
4	*	CCCCTTCTTGTCCTT	1.3	CTAGCTCCTTTGTCG	CAGAACGACAAAGCC
5	2.5	TGCCCCTCTTGTTCT	0.5	GCATTGTAGTGGCTG	CATACGAAGCACACC
6	*	CTCCTCTTCCTTGCT	-0.5	GGCATATAGCGTGAC	CCAGCCGATAACAAC
7	*	CTTCTCCCTTCCTCT	-0.4	GTTATTGCGACCTCG	GACCAACAGCAAGAC
8	*	CCTTCCTTCCCTCTT	-0.1	AGTCATGGACCAACG	CAAGCGTCATCCAAG
9	2.8	TCCCCTTGTGTGTGT	-2.4	GAACGGTTACCGATC	CACGCCATAAACCAG
10	-1.4	GAGAGAGAGGCCCCCTATCC	-0.6	AAAGACGTGTGAAGTGCGCT	CTACACTCTTCACTTCCACC
11	-2.2	GAAGAGAAGGGCACCCCTCC	-0.9	GACGAAAGTTCAGCAGCGAA	GCCTCATTCTTACCTCCTTC
12	0.6	GTGGTGTTGCGTCCCTTCCC	0.1	TGTTAAAATCAGGCTCGCGC	GAGACCGAAAGATAGCAGAG
13					CAACCGCTCAAATCTACTCC
					**GC ends**
				**#**	**§**
*L*_*c*_		10		5	5
*L*_*hp*_		5		5	2
*L*_*sc*_		6		6	4
*L*_*sl*_		8		4	5

Comparison of 15 and 20 bases long sequences generated by Arita et al. [13], Feldkamp et al. [20] and EGNAS. Molar free enthalpies of the most stable hairpins.

^*^No hairpin structure possible.

^#^Without {*GGG;CCC*}.

^§^Without {*GGG;CCC;AAAA;TTTT*}. For all sequences computed by EGNAS no possible hairpin structures could be found.

**Table 5:** Calculations with included sequences to design additional 15 bases long sequences

**No.**	**Δ*G***	**Faulhammer**	**Δ*G***	**Feldkamp**	**Δ*G***	**EGNAS**
	$\left[\frac{\mathrm{kcal}}{\mathrm{mol}}\right]$	**5’ → 3’**	$\left[\frac{\mathrm{kcal}}{\mathrm{mol}}\right]$		$\left[\frac{\mathrm{kcal}}{\mathrm{mol}}\right]$	**5’ → 3’**
1	*	ATCCTCCACTTCACA	*	CTTCTCTCACCTATA	*	CCTACAAATCAACTC
2	*	CTATTTCTCCACACC	*	GGCAAGAGGAATAAT	*	CTAAACACATCCAAC
3	*	CACCCTTTCTCCTCT	2.0	GCGAAAATTAACTCC	*	GCAGAACAAGATAAG
4	*	TCCTCACATTACTTA	1.9	GATCCGGTTACTAAA	*	CCTTCACTTACATTC
5	*	ACTTCCTTTATATCC	1.9	ACCTGACTCGTAATA	*	CTCTCACAATCTAAC
6	*	TCCACCAACTACCTA	1.6	TAAGTATATCGTGCC	*	CAATTTAACCTCCTC
7	2.6	AACTCTCAAATTCAA	1.6	GTCTGAGCTGATAAA	*	CTTCCATATACACTC
8	2.6	ACCTTACTTTCCATA	1.5	GTACCGTTGAATTGT	*	CCACACCTTAATATC
9	2.1	CTCTTACTCAATTCT	1.2	TGCGACTATGTTATG	*	CTATAATTCTCCACC
10	2.1	GTACATTCTCCCTAC	1.1	TTACAGCGTTTTACC	2.8	CGTTGTCTCTATTTC
11	1.8	TTATAACAAACATCC	1.0	AAAGCCGTCAAATAC	2.6	GTTCAGTATTCGTTC
12	1.8	TTTTAAATTTCACAA	1.0	TACCTTTTTGTCTCG	2.5	GTAGCGAAGAAAATG
13	1.6	ATAATCACATACTTC	0.8	ACAGGCGTATCTAAT	2.5	GGTTGCGTTTTATTG
14	1.6	CATTCCTTATCCCAC	0.4	AGTGACACTAGCATT	2.5	CATCGTCAAGTAAAG
15	1.4	CATATCAACATCTTA	0.4	ATGAGGCAGTCTTTA	2.5	CTTTGGTCTGTTATG
16	1.4	TTAAAATCTTCCCTC	0.2	AAGCTATTGATTGGC	2.2	GTCTTTTTGCTTTCG
17	1.3	CTAACCTTTACTTCA	0.1	CACTTGAGTACAACA	2.2	GCAGTTTCATAGTTC
18	1.2	GCTTCAAACAATTCC	0.1	GGATGTCCTTGTTTA	2.0	CTTCTACTACCTATC
19	1.2	ACATAACCCTCTTCA	-0.3	ACCAAACCATGATGA	1.9	GATTAGTGGTTTGAG
20	0.1	CATAATCTTATATTC	-0.7	TGGTAGGCCATTTAA	1.8	CTCATCATTACCATC
				**#**		**#**
						**GC ends**
*L*_*c*_		8		5		5
*L*_*hp*_		5		5		3
*L*_*sc*_		8		6		6
*L*_*sl*_		5		5		5

Comparison of 15 bases long sequences generated by Faulhammer et al. [14], Feldkamp et al. [20] and EGNAS. Molar free enthalpies of the most stable hairpins.

^*^No hairpin structure possible.

^#^Without {*GGG;CCC*}.

#### Calculations with included sequences

Another useful feature of a sequence design algorithm is the possibility to include already existing sequences into the calculations. As a result, the cross hybridization of newly formed sequences with the included strands is controllable. The EGNAS user may include naturally occurring sequences of genomic DNA, plasmids, cloning vectors, primers or previously designed sets of sequences with certain properties. For instance, we performed stepwise generation of sequences shown in Table 4. In the first step nine 15 bases long sequences were found. These sequences were included in the second run to design four additional 20 bases long sequences. With EGNAS, one more 20 bases long sequence was generated in comparison to sequences suggested by Arita et al. [13] and Feldkamp et al. [20]. This was possible in spite of extra restrictions according to GC ends and secondary structure. Possible hairpin structures could be completely eliminated in these computations.

The optimization of sequences is also possible with a different stepwise strategy. Here we first start with strong restrictions on sequence properties and weaken them until the desired set size is achieved.

Accordingly, the maximum stem length of hairpins were first limited to one base pair by setting *L*_*hp *_= 2. A set size of maximum 14 sequences was possible with this restriction. Finally, in the next sequence generation hairpins with stem length of 2 base pairs were allowed by setting *L*_*hp *_= 3 and guanine was forbidden. Through higher *L*_*hp*_, more variability of sequences is possible. Nevertheless, by forbidding guanine, there are only stem structures possible consisting of two consecutive adenine/thymine base pairs. These structures are less stable than those comprising guanine/cytosine base pairs. Therefore, other authors presented design algorithms that only use {*ATC*} for the sequence generation, and this aims to minimize the risk of hairpin formation [14,35]. In practice, this concept was realized for the TAG/anti-TAG system offered by Luminex^*Ⓡ*^[36]. For our calculations, the 14 previously generated sequences were included in order to expand the sequence set. Consequently, six further sequences could be generated. The result of this approach is shown in Table 5. It depicts a comparison between sets of 15 bases long sequences generated by Faulhammer et al. [14], Feldkamp et al. [20] and EGNAS. EGNAS offers better sequences with respect to secondary structures, as the minimum molar free enthalpies of the most stable hairpin structures are -0.7, 0.1 and 1.8 kcal/mol for Feldkamp et al., Faulhammer et al. and EGNAS, respectively. Comparing the criton length *L*_*c *_= 5 and the GC content of 40%, there is no improvement versus Feldkamp’s sequences. But a significant reduction of the secondary structure stabilities is evident, since the forbidden stem length was reduced from *L*_*hp *_= 5 to *L*_*hp *_= 3. Moreover, in contrast to Feldkamp’s sequences, only GC ends were allowed. In spite of this further restriction, an equal set size was achieved.

#### Balancing cross hybridization, sliding and hairpins

We investigated further advantages of discriminating between global criton rules and intrastrand properties. The results are summarized in Table 6. The generated sequences are compared with those of Shin et al. [16] and of Feldkamp et al. [20]. There are only seven 20 bases long sequences in one set. We show that the user of EGNAS can balance intra- and interstrand properties to achieve a required set size.

**Table 6:** Balancing cross hybridization, sliding and hairpins

**No.**	**Δ*G***	**Shinet al.**	**Δ*G***	**Feldkamp**
	$\left[\frac{\text{kcal}}{\text{mol}}\right]$	**5’ → 3’**	$\left[\frac{\text{kcal}}{\text{mol}}\right]$	**5’ → 3’**
1	0.8	AGGCGAGTATGGGGTATATC	1.0	TAGTCGCGTGATTTGGAAGG
2	0.8	TTATGATTCCACTGGCGCTC	0.6	TTACACTTGAAGCTGGCTCG
3	0.3	CTTCGCTGCTGATAACCTCA	0.3	CTTCGTGTCGGCCATCATAT
4	0.2	CGCTCCATCCTTGATCGTTT	-0.2	AAAGCCGTCGTTTAAGGAGC
5	0.1	ATCGTACTCATGGTCCCTAC	-0.3	GGTTCTTACGCTCTACTGCA
6	-0.3	GAGTTAGATGTCACGTCACG	-0.6	TACGTCTCGAACTGATAGCG
7	-2.3	CCTGTCAACATTGACGCTCA	-2.4	TCATGTTGGCACCGTATGCA
				#
*L*_*c*_		6		5
*L*_*hp*_		6		4
*L*_*sc*_		6		6
*L*_*sl*_		7		4
**No.**	**Δ*G***	**EGNAS**	**Δ*G***	**EGNAS**
	$\left[\frac{\mathrm{kcal}}{\mathrm{mol}}\right]$	**First set**	$\left[\frac{\mathrm{kcal}}{\mathrm{mol}}\right]$	**Second set**
		**5’ → 3’**		**5’ → 3’**
1	*	CAAAGAACCGACATAGCCAC	*	CCAACCAAACCACCAATCTC
2	*	GAACGGCAGGAGACAAATAC	1.4	CTGTCGTCGTGTCTTCTTCA
3	*	CATAAGAGGAAACAGCACGG	1.3	GCAGGCAGGTCAAGGTAAAT
4	2.1	GTTCGTCCTATTGCTCTGTG	1.0	ATCCGCCATAATAAGTCCGC
5	1.5	GTCGTGTTGCCTTTCTATCC	0.9	CTTTCGGCTCCTAACATTCG
6	1.3	GGTTTATTCTCGGCTTGTGG	0.7	GAGTGAGTTCCAGAGTATCG
7	1.3	GGCTCGTTTGGTGTATCTTC	0.5	TTGTAGCATCATCAGCGAGG
		**GC ends**		
		**#**		**#**
*L*_*c*_		5		4
*L*_*hp*_		2		3
*L*_*sc*_		4		4
*L*_*sl*_		3		6

Comparison of 20 bases long sequences generated by Shin et al. [16], Feldkamp et al. [20] and EGNAS. Molar free enthalpies of the most stable hairpins.

^*^No hairpin structure possible.

^#^Without {*GGG;CCC;AAAA;TTTT*}.

Because with *L*_*c *_= 5, *L*_*hp *_= 2 and *L*_*sl *_= 5, the set sizes clearly extended seven sequences, sliding could be decreased to *L*_*sl *_= 3 and even only GC ends were allowed for the first set. For the second set of sequences, we raised *L*_*sl*_ from 3 to 6, *L*_*hp*_ from 2 to 3 and lowered the criton length *L*_*c *_from 5 to 4. Consequently, we have to accept sliding of 5 bases long subsequences. Additionally, adenine or thymine had to be allowed as terminal bases to generate seven sequences. Indeed, this is against the global criton rule with *L*_*c *_= 5. However, no cross hybridization between different strands would take place with 4 bases long subsequences.

In both of our sets, the formation of hairpins is less probable then in the sets published by Shin et al. [16] and by Feldkamp et al. [20].

#### Computing time

In this section we demonstrate that the sequence generation lasts only a few seconds, if the user limits the set size reasonably. To this aim, 10 sets of 20 bases long sequences were generated for each set size with *L*_*c *_= 6 for a global criton rule. We varied the maximum number of sequences to be generated starting from 100. The highest defined set size was 109 sequences, since we know from calculations concerning the set size distribution that this is the minimum set size that could be generated for a complete set.

After the defined set size had been achieved, the sequence generation stopped and the computing time was saved. Insisting on the completeness of a set leads to considerable increase of the computing time from a few seconds (4 to 9 s) to approximately one minute (56.2 s ± 12.9 s). Nevertheless, this is still in reasonable computing time for a complete set size. Actually, the computing time grows considerably with *L*_*c*_. The generation of a complete set with *L*_*c *_≥ 8 can last hours, as the number of basic sequences also grows exponentially with *L*_*c*_(Equations (1) and (2)).

### Combination of TAGs with primers

**Figure 6:**
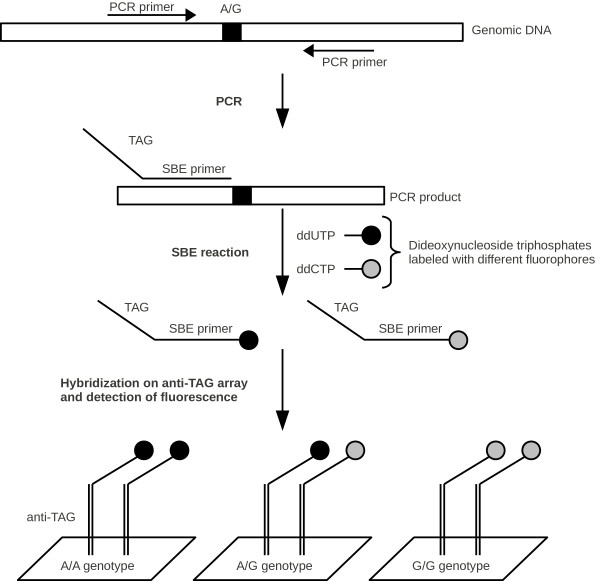
**Principle of a TAG-SBE genotyping assay.** Principle of a TAG-SBE genotyping assay to detect an A/G SNP. TAG sequences were designed and paired with primers by using EGNAS.

Genotyping of single nucleotide polymorphisms (SNPs) is one example for a particular application of SBE reactions (Figure 6). This technique, as described by Fan et al., requires SBE-TAG primers [8]. Marker-specific polymerase chain reaction (PCR) primers are needed for the amplification of SNPcontaining regions. Thereafter, the PCR products are used as templates for the SBE reaction with SBE-TAG primers. The 3’ ends of these primers are complementary to the specifically flanking regions of the SNP loci and the 5’ ends are complementary to specific probes on an array. The generation of suitable SBE-TAGs was performed with the EGNAS algorithm. In this case, the TAG sequences were generated with respect to minimal interaction with the neighboring SNP flanking sequence. Consequently, no 3 bases long subsequences were complementary between one SNP flanking sequence and its corresponding TAG sequence (*L*_*ni *_= 3). The primers and SNP flanking sequences were taken from Fan et al. [8] to generate 148 TAGs that were 20 bases long with *L*_*c *_= 8, *L*_*sl *_= 8, *L*_*hp *_= 2 and GC content 50%. The subsequences {*GGG*;*CCC*;*AAAAA*;*TTTTT*} were forbidden and GC ends were demanded. Forward and reverse primers as well as the flanking regions of the SNPs were chosen as included sequences. Thus, no cross hybridizations with 8 bases long subsequences occur among the TAGs and included sequences, secondary structures were avoided within the TAGs and foldback due to interactions with SNP flanking sequences were minimized.

We generated TAGs without stable hairpins (*L*_*hp *_= 2, Δ*G *> 0.4 kcal/mol) by using EGNAS. In contrast, the TAGs presented by Fan et al. show one very stable hairpin with a stem length of 3 base pairs (*L*_*hp *_= 4, Δ*G *=−2.7 kcal/mol). Moreover, with regard to foldbacks, the TAG-primer pairing with EGNAS is better (*L*_*ni *_= 3) than presented by Fan et al. (*L*_*ni *_= 7) for 148 SBE-TAG primers. The advantages of EGNAS become also apparent by comparing the uniqueness of the TAG sequences. Fan et al. used TAGs with *L*_*c *_= 11 and EGNAS provided TAGs with *L*_*c *_= 8. Thus, the quality of the TAGs can be evaluated by the values of *L*_*hp*_, *L*_*ni *_and *L*_*c*_, when hairpins, TAG-primer pairing and cross hybridization are in the focus, respectively. Corresponding results are shown in Tables 7 and 8. All TAGs and primer sequences as well as the molar free enthalpies of the most stable hairpins are given in additional files [see Additional file 5 and Additional file 6].

**Table 7:** Combination of TAGs with Primers — comparison of TAGs

	**TAGs**	
	**Fanet al.**	**EGNAS**^1*^
Average Δ*G*	-0.3 ± 0.9	1.6 ± 0.5
Minimum Δ*G*	-2.7	0.4
GC content	45% - 50%	50%
*L*_*c*_	11	8
*L*_*hp*_	4	2
*L*_*sc*_	6	4
*L*_*sl*_	7	8
*L*_*ni*_	7	3

Comparison of TAGs used by Fan et al. [8] and those generated by EGNAS. Intra- and interstrand properties. Molar free enthalpies (Δ*G*, kcal/mol) of the most stable hairpins. Averages ± standard deviations from 148 sequences.

^*^GC ends and without {*GGG;CCC;AAAAA;TTTTT*}.

### TAGs for staple strands of DNA origami

Self-assembled structures are fundamental in the field of nanotechnology. DNA allows the assembly of programmable building blocks. One promising method to control and organize functional materials is based on DNA origami [37]. The underlying concept requires an accurate design of DNA sequences. A spatially precise functionalization is accessible through the unique addressability of DNA origami on the nanometer scale. The DNA origami scaffold is a long single strand of DNA, which is folded by hundreds of short synthetic oligonucleotides called staple strands. The staple strands are designed to bind several desired parts of the scaffold, and thus, to connect otherwise distant sites of this long single-stranded DNA. Rothemund presented a variety of different shapes based on this concept [6].

There is software available for the design of 3D DNA origami shapes, for instance the caDNAno software [38]. However, we show that EGNAS will be useful to find proper sequences, if staple strands have to be extended by anti-TAGs that stay single-stranded during origami formation. These anti-TAGs are used as sticky ends or capture probes to bind the TAGs of DNA-modified nanoparticles or peptide-DNA conjugates to a given DNA origami structure. In the following, we examine two examples with single-stranded circular M13mp18 viral DNA as scaffold. Its sequence [39] was included in the calculations below.

#### Sticky ends for triangular DNA origami

**Figure 7:**
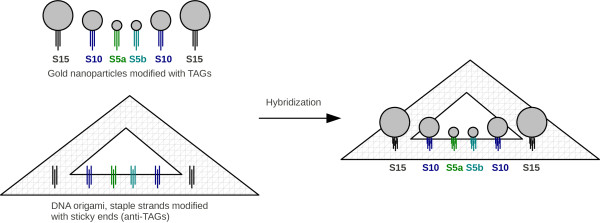
**Principle of the arrangement of gold nanoparticles on a triangular DNA origami.** Principle of the arrangement of gold nanoparticles on a triangular DNA origami by extending staple strands with sticky ends. The corresponding sequences of the sticky ends were designed and paired by using EGNAS.

In the work of Ding et al., triangular DNA origami is used for the assembly of six gold nanoparticles through DNA hybridization (Figure 7) [40]. The particles have sizes of either 5, 10 or 15 nm. Each particle is captured by three probes on the DNA origami with the M13mp18 DNA as a scaffold. To this aim, 18 staple strands are modified with 24 bases long sticky ends (anti-TAGs). Ding et al. designed four different TAG sequences to label the 10 and 15 nm gold particles each with one sequences, and to modify two differently labeled 5 nm particles.

**Table 8:** Combination of TAGs with Primers — TAG-primer pairing

	**Flanking**	**TAG-primer pairs**	
	**sequences**	**Fanet al.**	**EGNAS**
Average Δ*G*	-0.7 ± 1.2	-2.8 ± 1.4	-1.2 ± 1.1
Minimum Δ*G*	-4.3	-9.0	-4.3

Comparison of SBE-TAGs used by Fan et al. [8] and those generated by EGNAS. Molar free enthalpies (Δ*G*, kcal/mol) of the most stable hairpins. Averages ± standard deviations from 148 sequences.

**Table 9:** Modification of staple strands for DNA origami

	**Triangular DNA origami**		
	**Not yet modified**	**Modified staple strands**	
	**staple strands**	**Dinget al.**	**EGNAS**
Average Δ*G*	-1.5 ± 1.4	-3.2 ± 1.7	-2.2 ± 1.3
Minimum Δ*G*	-5.2	-6.8	-5.2
	**Six-helix bundle nanotubes**		
	**Not yet modified**	**Modified staple strands**	
	**staple strands**	**Stearnset al.**	**EGNAS**
Average Δ*G*	-1.36 ± 1.1	-5.9 ± 1.3	-1.8 ± 1.4
Minimum Δ*G*	-3.0	-8.0	-4.2

Comparison of sequences that were generated by using EGNAS, and those used by Ding et al. [40] or Stearns et al. [41] to modify staple strands of triangular DNA origami or six-helix bundle nanotubes, respectively. Molar free enthalpies (Δ*G*, kcal/mol) of the most stable hairpins. Averages ± standard deviations.

We analyzed possible cross hybridization of the sticky ends with the staple strands and with the scaffold. As a result, the 9 bases long sequence 5’-GAATCCTGA-3’ is identical in the staple strand “C28” and in the TAG “S5a” of the 5 nm gold nanoparticles. This could result in a cross hybridization of three modified staple strands with the unmodified “C28” strand. There are also numerous 8 and 9 bases long sequences that could cause cross hybridizations with the scaffold strand. Additionally, we found possible hairpin structures with a 4 base pairs long stem (5’-AGTC-3’) in the sequences of the TAG “S5b” and the corresponding anti-TAG. Hairpin structures lower the hybridization efficiency of the TAGs and anti-TAGs. This could be one explanation for missing 5 nm particles on the DNA origami. Actually, Ding et al. mentioned this problem when only two sticky ends per particle were used.

We tried to improve the sequences of the sticky ends by applying EGNAS. To this aim, we included the scaffold and all staple strand sequences in the calculations. More precisely, we chose *L*_*c *_= 8, and thus, avoided cross hybridizations with more than 7 consecutive base pairs of the sticky ends with the modified and unmodified staple strands, as well as with the scaffold. Secondary structures were evaded by setting *L*_*hp *_= 2. We generated the sticky ends stepwise and included the previously designed sequences of the modified staple strands for the design of the next sticky end. Furthermore, all 4 bases long sequences that were partly complementary to the neighboring staple strands were forbidden for the current generation of the corresponding sticky end (*L*_*ni *_= 4). The risk of foldbacks is minimized by that procedure.

Consequently, the sequences used by Ding et al. to modify staple strands exhibit the possibility of more stable cross hybridizations and secondary structures compared with sequences that were generated with EGNAS (Table 9). The sequences of staple strands before and after modification as well as their molar free enthalpies of the most stable hairpins are given in an additional file [see Additional file 7].

#### Capture probes for six-helix bundle nanotubes

**Figure 8:**
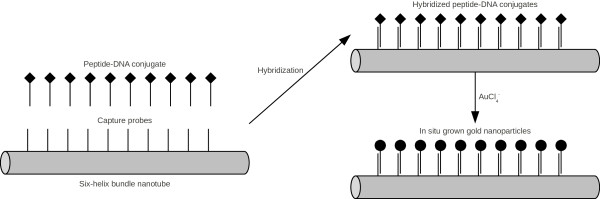
**Principle of in situ nucleation and growth of gold nanoparicles on a six-helix bundle nanotube.** Principle of in situ nucleation and growth of gold nanoparicles on a six-helix bundle nanotube with extended staple strands. The corresponding sequence of a capture probe was designed and paired by using EGNAS.

Stearns et al. tried to organize a peptide-DNA conjugate on a six-helix bundle forming DNA origami (Figure 8) [41]. The peptide A3 was used for in situ nucleation and growth of gold nanoparticles, as it recognizes gold surfaces and reduces soluble gold ions. Only one sequence for capture probes was used to modify 10 staple strands to fold the M13mp18 DNA as a scaffold. Again, we analyzed the cross hybridization of the probe sequence with the staple strands and with the scaffold. Only one 8 base pairs long possible cross hybridization with the sequence 5’-GCCGTTGA-3’ of the staple strand “70” with the peptide-DNA conjugate was found. There are possible hairpin structures with a 5 base pairs long stem (5’-CGTTG-3’) in the probe sequences. Furthermore, foldback structures are possible through 5 bases long complementary subsequences (5’-AACGG-3’) of the probe and the staple strand “122”.

Here, we optimized the probe sequence with equal parameters like presented above for the sticky ends for triangular DNA origami. As a result, to avoid secondary structures, the sequences that were generated by using EGNAS are more suitable to capture the peptide-DNA conjugate compared with those used by Stearns et al. to modify the staple strands (Table 9). Sequences of staple strands before and after modification as well as their molar free enthalpies of the most stable hairpins are given in an additional file [see Additional file 8]. Stearns et al. reported that the particles were not organized in the designed 27 nm intervals. Hence, the peptide was probably immobilized not efficiently enough. Hairpin formation of the probe and foldback with the staple strands might be the reasons for the low density of nanoparticles on the DNA origami.

## Conclusions

We developed a new software tool called EGNAS for the design of unique nucleic acid sequences. Sets of sequences with defined intra- and interstrand properties can be generated in reasonable computing time. A maximum set size with given constraints can be achieved. The presented exhaustive algorithm allows to generate greater sets of sequences than with previous software and equal constraints.

In the present stage, the developed program is suitable for generating sequences for DNA-TAGs avoiding secondary structures and cross hybridizations. Furthermore, predefined sequences can be taken into consideration. This is in principle useful for applications where no interaction of TAGs with template strands is desired. The offered option of TAG-primer pairing with regard to minimal foldback facilitates the generation of TAG sequences for multiplexed genotyping of SNPs. This kind of genotyping can be performed with PCR and SBE reactions on microarrays or bead surfaces. Additionally, EGNAS affords the computer aided design of sequences for specific attachment of molecular constructs to DNA origami. In further development the novel algorithm could be optimized to efficiently include large gene sequences. An extension of EGNAS to design branched structures like nucleic acid junctions is possible.

## Availability and requirements

**Project name:** EGNAS

**Project home page:**http://www.chm.tu-dresden.de/pc6/EGNAS

**Operating systems:**Linux, Mac OS X, and Microsoft Windows

**Programming language:** C++

**Other requirements:** None

**License:** Free for noncommercial use

**Any restrictions to use by nonacademics:** License needed

## Abbreviations

EGNAS: Exhaustive generation of nucleic acid sequences; DNA: Deoxyribonucleic acid; A: Adenine; T: Thymine; G: Guanine; C: Cytosine; GC: Content, Guanine-cytosine content; SBE: Single-base extension; SNP: Single nucleotide polymorphism; PCR: Polymerase chain reaction; ddUTP: Dideoxyuridine triphosphate; ddCTP: Dideoxycytosine triphosphate.

## Competing interests

The authors declare that they have no competing interests.

## Authors’ contributions

AK conceived the algorithm, performed the calculations and drafted the manuscript. MB participated in developing the algorithm and wrote the software program in C++. MM helped to draft the manuscript and revised it critically. All authors read and approved the final manuscript.

## Supplementary Material

Additional file 1 **Manual for the use of EGNAS.** This DOC file contains the manual for the use of EGNAS. The sequence design criteria and options are explained [27,28]. Click here for file

Additional file 2 **Executable file for Linux operating systems.** This ZIP file contains the executable file of EGNAS for Linux operating systems. Click here for file

Additional file 3 **Executable file for Mac OS X operating systems.** This ZIP file contains the executable file of EGNAS for Mac OS X operating systems. Click here for file

Additional file 4 **Executable file for Microsoft Windows operating systems.** This ZIP file contains the executable file of EGNAS for Microsoft Windows operating systems. Click here for file

Additional file 5 **SBE-TAGs and primer sequences.** This Microsoft Office Excel 2003 sheet contains SBE-TAGs and primer sequences as well as the molar free enthalpies of the most stable hairpins. Click here for file

Additional file 6 **SNPs and PCR primer sequences.** This Microsoft Office Excel 2003 sheet contains SNPs and PCR primer sequences as well as the molar free enthalpies of the most stable hairpins. Click here for file

Additional file 7 **Sequences for triangular DNA origami.** This Microsoft Office Excel 2003 sheet contains the sticky end sequences for triangular DNA origami. The sequences of staple strands are shown before and after modification. Their molar free enthalpies of the most stable hairpins are presented. Click here for file

Additional file 8 **Sequences for DNA origami forming six-helix bundle nanotubes.** This Microsoft Office Excel 2003 sheet contains the capture probe sequences for DNA origami forming six-helix bundle nanotubes. The sequences of staple strands are shown before and after modification. Their molar free enthalpies of the most stable hairpins are presented. Click here for file
