# Supplementary material for: EGNAS: an exhaustive DNA sequence design algorithm
Source: BMC Bioinformatics. 2012 Jun 20;13:138. doi: 10.1186/1471-2105-13-138 (PMC3496572; doi:10.1186/1471-2105-13-138)
Supplement: Additional file 1 — Manual for the use of EGNAS. This DOC file contains the manual for the use of EGNAS. The sequence design criteria and options are explained [27,28]. [file 1471-2105-13-138-S1.doc]

**EGNAS software manual**

# Running the program

The sequence design algorithm EGNAS is realized in a program written in C++. It is currently a command line program which was compiled for Linux, Mac OS X and Microsoft Windows operating systems.

EGNAS is started by running the executable file after unpacking the ZIP file for the proper operating system.

Forbidden subsequence motifs can be given in a text file named “forbidden.txt”. This file and the text files with the included and neighboring sequences have to be saved in the folder of the executable file. All of those sequences have to be separated by a newline and consist of the characters from the set {A;T;C;G}. Additionally, a configuration file is needed to choose the sequence design criteria and options. If no configuration file “config.txt” exists in the current folder, the program will create the “config.txt”.

# Sequence design criteria and options

The sequence design algorithm EGNAS offers the user different options. Consequently, the generated sequences meet certain criteria:

1. Sequence length Ls.
2. Length of basic sequences (criton length) L_c.
3. Exact GC content or its range.
4. No terminal adenine or thymine in the strand./Demand on “GC ends”.
5. Forbidden sequences./Included sequences.
6. Length of forbidden self-complementary subsequences L_sc.
7. Forbidden stem length of hairpin structures L_hp.
8. Length of subsequences that are not allowed to be repeated within one and the same sequence (“sliding”) L_sl.
9. Forbidden length of subsequences that could interact with complementary neighboring sequences L_ni.

The EGNAS software provides the option to calculate the molar free enthalpy of DNA duplex formation. This calculation is based on the nearest-neighbor model [27] with parameters taken from SantaLucia et al. [28].

# The configuration file “config.txt”

The configuration file “config.txt” contains the settings for the sequence design. An example is shown below:

#Created with version: 1158

#Sequence length L_s.

L_s[bases]=24

#Length of basic sequences (criton length) 1 <= L_c <= 14.

L_c[bases]=5

#Forbidden stem length of hairpin structures L_hp (blank for any size).

L_hp[base pairs]=3

#Length of forbidden self-complementary subsequences L_sc (blank for any size).

L_sc[bases]=4

#Minimum number of G/C-bases per strand GC_min, minimum GC content (blank for

any number).

GC_min[bases]=11

#Maximum number of G/C-bases per strand GC_max, maximum GC content (blank for

any number).

GC_max[bases]=13

#Length of subsequences that are not allowed to be repeated within one and the

same sequence (sliding) L_sl (blank for any size).

L_sl[bases]=5

#No terminal adenine or thymine in the strand?/Demand on GC ends? [y/n]

GC_ends=n

#Maximum number of sequences to be generated per set.

SeqMax=1250

#Maximum number of sets to be generated.

SetMax=2

#Maximum number of attempts to restart the generation of one sequence.

MaxAttempt=50

#Termination factor to limit the number of combination attempts with basic

sequences for the generation of one strand.

TerminationFactor=1

#Name of the file with sequences to be included (blank, if no file exist).

Included=

#Name of the file with neighboring sequences (blank, if no file exist).

Neighboring=

#Forbidden stem length of hairpin structures with neighboring sequences L_ni

(neighbor interaction).

L_ni[base pairs]=

#Calculate deltaG? [y/n]

deltaG=n

# The output of the results

The output of the results is saved in a separate folder. Its name is the current date and system time. A log file contains the settings and further information about the sequence generations. Every generated set of sequences is saved in a separate text file.

# Availability and requirements

Project name: EGNAS

Project home page: http://www.chm.tu-dresden.de/pc6/EGNAS

Operating systems: Linux, Mac OS X, and Microsoft Windows

Programming language: C++

Other requirements: None

License: Free for noncommercial use

Any restrictions to use by nonacademics: License needed
